# The comparison between fixed versus degressive doses of medroxyprogesterone acetate combined with letrozole in patients of progestin-primed ovarian stimulation protocol: a propensity score-matched study

**DOI:** 10.3389/fendo.2023.1295787

**Published:** 2023-12-12

**Authors:** Ying Zhang, Hao Li, Shanshan Zhu, Shengfang Jiang, Wenxian Zhao, Xiaoning Wang, Liu Tian, Guangming Zhao, Nongqiao He, Honglu Diao, Hong Cao, Changjun Zhang

**Affiliations:** ^1^ Reproductive Medicine Center, Renmin Hospital, Hubei University of Medicine, Shiyan, China; ^2^ Hubei Clinical Research Center for Reproductive Medicine, Shiyan, China; ^3^ Biomedical Engineering College, Hubei University of Medicine, Shiyan, China; ^4^ Biomedical Research Institute, Hubei University of Medicine, Shiyan, China; ^5^ Hubei Key Laboratory of Embryonic Stem Cell Research, Hubei University of Medicine, Shiyan, China; ^6^ The Third Medical School, Hubei University of Medicine, Shiyan, China; ^7^ Department of Orthopaedic Surgery, Renmin Hospital, Hubei University of Medicine, Shiyan, China

**Keywords:** progestin primed ovarian stimulation, medroxyprogesterone acetate, dose reduction, controlled ovarian stimulation, letrozole

## Abstract

**Objective:**

To explore the cycle characteristics and pregnancy outcomes of progestin-primed ovarian stimulation (PPOS) using fixed versus degressive doses of medroxyprogesterone acetate (MPA) in conjunction with letrozole (LE) in infertile women by propensity score matching (PSM) analysis.

**Design:**

A retrospective cohort study.

**Setting:**

Tertiary-care academic medical center.

**Population:**

A total of 3173 infertile women undergoing their first *in vitro* fertilization/intracytoplasmic sperm injection (IVF/ICSI) treatment within the period from January 2017 to December 2020.

**Methods:**

A total of 1068 and 783 patients who underwent a fixed dose of MPA combined with LE and a degressive dose of MPA combined with LE protocols, respectively, were enrolled in this study. The freeze-all approach and later frozen-thawed embryo transfer (FET) were performed in both groups. Propensity score matching (1:1) was performed.

**Main outcome measures:**

The primary outcomes were the dosage of MPA and the incidence of premature luteinizing hormone (LH) surges. The secondary outcomes were the number of oocytes retrieved, the cumulative live birth rate (CLBR) and the fetal malformation rate.

**Results:**

We created a perfect match of 478 patients in each group. The dosage of MPA, the LH serum level on the eighth day of stimulation, progesterone (P) level and LH level on the hCG trigger day were significantly higher in the LE + fixed MPA group than in the LE + degressive MPA group (52.1 ± 13.1 mg vs. 44.9 ± 12.5 mg; 5.0 ± 2.7 IU/L vs. 3.7 ± 1.7 IU/L; 0.9 ± 0.5 ng/ml vs. 0.8 ± 0.5 ng/ml; 3.3 ± 2.4 IU/L vs. 2.8 ± 1.9 IU/L; *P* < 0.01). The duration of Gn, the number of follicles with diameter more than 16 mm on trigger day, the estradiol (E_2_) level on the hCG trigger day were lower in the LE + fixed MPA group than in the LE + degressive MPA group (9.7 ± 1.7 days vs. 10.3 ± 1.5 days; 5.6 ± 3.0 vs. 6.3 ± 3.0; 1752.5 ± 1120.8 pg/ml vs. 1997.2 ± 1108.5 pg/ml; *P* < 0.001). No significant difference was found in the incidence of premature LH surge, the number of oocytes retrieved, the number of top-quality embryos, clinical pregnancy rate (CPR), CLBR or fetal malformation rate between the two groups.

**Conclusion:**

The combination of a degressive MPA dose with LE proved effective in reducing the total MPA dosage with comparable premature LH surge and pregnancy outcomes in women undergoing the PPOS protocol.

## Introduction

The progestin-primed ovarian stimulation (PPOS) protocol has become widely used in *in vitro* fertilization/intracytoplasmic sperm injection (IVF/ICSI) treatments as an alternative to gonadotropin-releasing hormone (GnRH) analog protocols for inhibiting premature luteinizing hormone (LH) surges (1, 2). This protocol offers several advantages, making it a favored option in clinical practice. First, it can be administered orally, which is highly convenient for patients. Second, it is more cost-effective than other controlled ovarian hyperstimulation (COH) protocols. Third, the PPOS protocol is associated with shorter treatment durations, saving time for both patients and healthcare providers. Most importantly, it significantly reduces the occurrence of ovarian hyperstimulation syndrome (OHSS), a severe complication associated with other COH protocols. Due to these benefits, the PPOS protocol is considered suitable for women with various ovarian responses, including those with poor ovarian response (3–6), normal responders (7, 8), and even high responders (7, 9) in IVF/ICSI cycles.

Since its introduction in 2015 (10), the PPOS protocol has been subject to various progestin administration investigations, with medroxyprogesterone acetate (MPA) being the most commonly used. MPA is a potent synthetic progestin that effectively suppresses pulsatile GnRH and LH secretion. Previous research has shown that 10 mg of MPA effectively inhibits spontaneous ovulation, whereas 5 mg does not yield the same results (11). However, conflicting findings have been reported regarding the appropriate MPA dosage for preventing untimely LH surges, with some studies suggesting that daily doses of 4 mg (12, 13) or 6 mg (3, 6) are sufficient. In our previous study, we demonstrated that coadministration of letrozole (LE) with MPA during ovarian stimulation for IVF achieved comparable embryo and pregnancy outcomes while reducing the required MPA dosage (14). Nonetheless, it is crucial to address the potential teratogenicity and toxicity associated with MPA, as several human and animal studies have indicated a dose-related relationship (15–20). As a result, we have been exploring avenues to reduce the MPA dose while maintaining its inhibitory effect and ensuring the safety of the PPOS protocol.

Hence, we hypothesized that coadministration of LE with a degressive dose of MPA based on serum LH levels may offer the potential for further reducing the required MPA dosage. The objective of this retrospective cohort study was to investigate the effects of this degressive MPA dose combined with LE on cycle characteristics, endocrinological profiles, and neonatal outcomes in IVF/ICSI cycles.

## Materials and methods

### Study setting and subjects

We conducted a hospital-based retrospective cohort study, adhering to the principles outlined in the Declaration of Helsinki, and obtained approval from the Ethics Committee of Renmin Hospital, Hubei University of Medicine. The data were collected from the Reproductive Medicine Center, Renmin Hospital, Hubei University of Medicine, covering the period from January 2017 to December 2020. All data collected were anonymized to ensure patient confidentiality and privacy.

**Figure 1:**
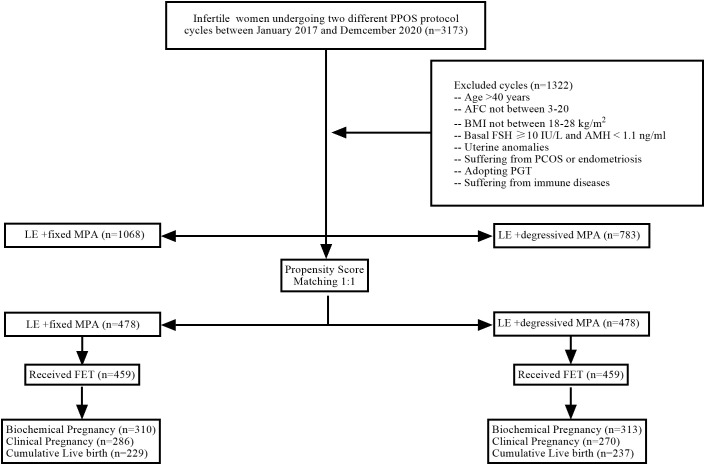
Flow chart of patient inclusion/exclusion. PPOS, progestin-primed ovarian stimulation; n, number of participants; AFC, antral follicle count; BMI, body mass index; FSH, follicle-stimulating hormone; AMH, anti-Müllerian hormone; PCOS, polycystic ovarian syndrome; PGT, preimplantation genetic diagnosis/screening; LE, letrozole; MPA, medroxyprogesterone acetate; FET, frozen embryo transfer.

Patients who underwent the PPOS protocol were included in the study if they met the following criteria: women with regular menstrual cycles ranging from 25 to 35 days, aged between 20 and 40 years, and had a body mass index (BMI) between 18 and 28 kg/m^2^. Additionally, bilateral antral follicle counts (AFCs) were required to be between 3 and 20, and normal basal serum levels of follicle-stimulating hormone (FSH) (<10 IU/L) and anti-Müllerian hormone (AMH) (≥1.1 ng/ml) were determined on day 2 or 3 of the cycle before COH. Study exclusion criteria included patients with metabolic disorders, polycystic ovarian syndrome (PCOS), endometriosis, pelvic tuberculosis, congenital uterine malformations, chromosomal abnormalities, single-gene disorders, and immunological diseases (**Figure 1**). Pregnancy outcomes were followed through telephone contact with the participants.

### Controlled ovarian stimulation

All patients received an ultrasound scan and serum concentration tests on day 2 or 3 of the cycle.

**Figure 2:**
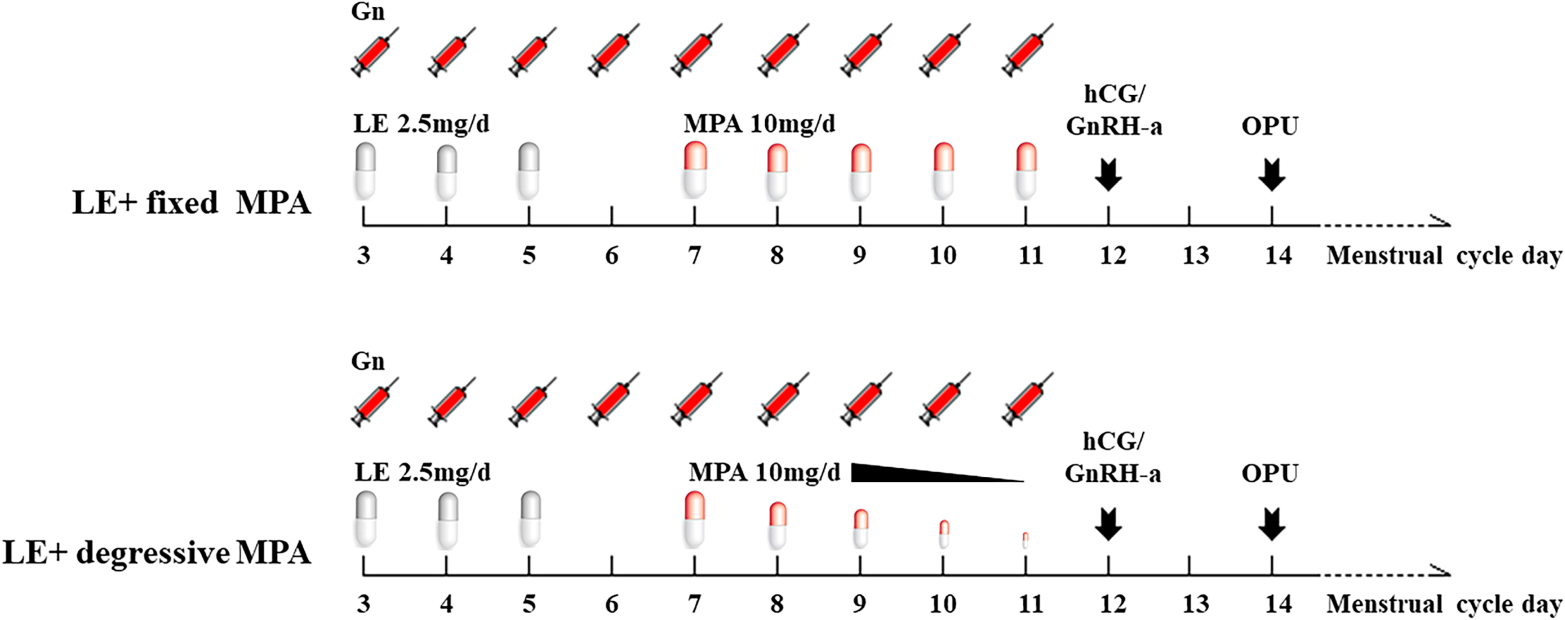
The diagram of the two PPOS protocols. LE, letrozole; MPA, medroxyprogesterone acetate; Gn, gonadotropin; hCG, human chorionic gonadotropin; GnRH-a, gonadotropin-releasing hormone agonist; OPU, oocyte pick-up.

In the LE+ fixed MPA group, oral LE (Jiangsu Hengrui Pharmaceuticals Co.,Ltd, China) 2.5 mg/day was started on day 2 or 3 of menstruation for 3 days, along with gonadotropin (Gn) stimulation of recombinant FSH (Gonal-f, Merck Serono, Germany) 100-150 IU/day intramuscularly, and the doses of urinary human menopausal gonadotropin (HMG, Livzon Pharmaceutical, China) and recombinant FSH were adjusted according to the growth trend of the follicles and serum hormone changes (150-450 IU per day). MPA (Zhejiang Xianju Pharmaceutical Co., China) 10 mg/day was started on day 5 of Gn use and stopped on the trigger day. Triptorelin (Decapeptyl, Ferring Pharmaceuticals, Germany) at a dose of 0.1 mg and urinary hCG (Livzon Pharmaceutical, China) at a dose of 2,000 IU were given to trigger oocyte maturation when two or more follicles reached preovulatory size (18-22 mm). Oocyte retrieval was performed 36 hours after the trigger (**Figure 2**). According to the standard insemination procedures used in the laboratory, all oocytes were inseminated using IVF or ICSI. Embryo scoring was conducted based on morphologic criteria; 6-8 cells with less than 20% fragmentation were considered good-quality embryos. These embryos were cultured forward when the number equaled or was more than three until they reached the blastocyst stage and were frozen on day 5 or day 6.

In the LE+ degressive MPA group, MPA 10 mg/day was started on day 5 of Gn use, and then, the dosage of MPA was gradually reduced if the serum LH level did not increase (**Figure 2**). We used 10 mg per day when the LH level increased to more than 10 IU/L in the process of stimulation. The other treatments were the same as above.

### Hormonal measurement

Serum FSH, LH, estradiol (E_2_), and progesterone (P) were measured on day 3 of the stimulation cycle (first day of stimulation), cycle day 6 (fourth day of stimulation), cycle day 8 (sixth day of stimulation), cycle day 10 (eighth day of stimulation), hCG trigger day, and the day after hCG trigger (approximately 12 hours after the injection of GnRH-a and hCG). Hormone levels were measured with electrochemiluminescence (Beckman Coulter, USA). Skilled technicians carried out all measurements in accordance with the manufacturer’s instructions. The detection limits of sensitivity were as follows: FSH, 0.2 IU/L; LH, 0.2 IU/L; E_2_, 15 pg/ml; and P, 0.1 ng/ml. The in-house inter and intra-assay coeffients of variation were no more than 10%.

### Endometrial preparation and frozen-thawed embryo transfer

Endometrial preparation was performed with natural cycle, hormone replacement treatment (HRT) or downregulation combined with HRT for the second cycle after oocyte retrieval. The decision of the therapy was determined according to patient and physician preference.

In the natural cycle, the follicle growth was exanimated by transvaginal ultrasound from day 10 of menstruation per 2 days till ovulation happened, then luteal-phase support was initiated with 10 mg twice oral dydrogesterone (Duphaston, Abbott, USA) and continued daily until 3 months of gestation.

In the HRT cycle, women were administered 2 mg twice oral estradiol valerate tablets (Progynova, Berlin, Germany) on day 3 of spontaneous menses or P-induced withdrawal bleeding. The dosage of Progynova was adjusted according to the endometrial thickness and serum E_2_ levels, and the maximum dose was 8 mg per day. After 16 days, when the endometrial thickness reached ≥ 7 mm and the serum concentration of E_2_ was ≥ 100 pg/ml, luteal-phase support was initiated with the application of 90 mg vaginal progesterone gel (Crinone; Merck Serono) or 60 mg intramuscular progesterone (Zhejiang Xianju Pharmaceutical Co., China) and 10 mg twice oral dydrogesterone.

In the downregulation combined with HRT cycle, the patients received a single intramuscular injection of 3.75 mg long-acting triptorelin acetate (Decapeptyl; Ferring, SaintPrex, Switzerland) on day 3 of the cycle. After 35 days of downregulation, oral estradiol valerate tablets were added, and the other procedure was the same as above.

## Outcome measurements

### Primary outcomes

The primary outcomes were the dosage of MPA and the incidence of premature luteinizing hormone (LH) surges. The premature LH surge was defined as serum LH > 10 IU/L during stimulation. Viable embryos were estimated based on embryo morphologic scoring conducted on day 3 after oocyte retrieval.

### Secondary outcomes

Secondary efficacy parameters include the number of oocytes retrieved, the cumulative live birth rate (CLBR) and the fetal malformation rate from a single IVF cycle. The endpoint was cumulative live birth or the use of all embryos.

Moderate/severe OHSS was diagnosed in women who fulfilled more than one of the following criteria: clinical ascites, hydrothorax, or dyspnea (exertional or at rest). Biochemical pregnancy was defined as hCG >10 IU/L two weeks after embryo transfer (ET). Clinical pregnancy was defined as an intrauterine gestational sac identified by ultrasonography 30 days after ET. Early pregnancy loss was defined as spontaneous pregnancy loss before 12 weeks. Live birth was considered when a living fetus was born after 28 weeks of pregnancy. CLBR was calculated as the number of live birth cycles/total number of oocyte retrieval cycles.

### Statistical analysis

All analyses were performed using the statistical packages R (The R Foundation; http://www.r-project.org; version 3.4.3), EmpowerStats (http://www.empowerstats.com) and SPSS 26.0 (IBM, Armonk, NY, USA). Continuous variables were presented as mean with standard deviation or median with interquartile range, and one-way analysis of variance or Kruskal–Wallis test was used to compare the differences among groups. Categorical variables were described as number with percentage and compared by Pearson’s chi-square test or Fisher’s exact test. We constructed a multivariable regression model to quantify the related factors of pregnancy outcomes in all participants. Statistical significance was accepted as a two-sided *P* value < 0.05. Graphs were generated by using Originpro 2018C version 9.5.1.195 (Originlab).

## Results

### Patient characteristics

From the initial cohort of 3,173 IVF/ICSI cycles, 1,322 cycles were excluded from the analysis. After the exclusions, the eligible cohort included 1,068 women using the LE+ fixed MPA protocol, 783 women using the LE+ degressive MPA protocol, and 478 patients in each group when propensity score matching (PSM) was performed (**Table 1**). There were no statistically significant differences in female age, BMI, AFC, AMH, infertility duration, infertility type, or infertility diagnosis between the two groups (*P* > 0.05) (**Table 1**).

**Table 1:** Baseline characteristics of the two PPOS protocols.

	Before propensity matching			After propensity matching		
	LE+ fixed MPA	LE +degressive MPA	*P*-value	LE+ fixed MPA	LE +degressive MPA	*P*-value
No. of cycles	1068	783	/	478	478	/
Female Age (years)	(1068) 33.0 ± 4.2	(783) 32.6 ± 4.2	0.032	(478) 32.4 ± 4.1	(478) 32.4 ± 4.2	0.957
BMI (kg/m^2^)	(1068) 22.4 ± 2.4	(783) 22.9 ± 2.5	<0.001	(478) 22.7 ± 2.5	(478) 22.6 ± 2.4	0.370
AFC	(1068) 7.5 ± 3.3	(783) 7.8 ± 3.3	0.033	(478) 7.8 ± 3.4	(478) 7.7 ± 3.2	0.860
AMH (ng/ml)	(1068) 2.5 ± 1.8	(783) 2.4 ± 1.9	0.734	(478) 2.5 ± 1.8	(478) 2.5 ± 2.1	0.964
Infertility duration (years)	(1068) 4.1 ± 3.3	(783) 3.8 ± 3.1	0.112	(478) 3.8 ± 2.9	(478) 4.0 ± 3.0	0.491
Primary Infertility n (%)	846/1068 (79.2%)	773/783 (98.7%)	<0.001	460/478 (96.2%)	468/478 (97.9%)	0.125
Infertility diagnosis, n (%)			0.094			0.587
Tubal factor	475/1068 (44.5%)	358/783 (45.7%)		226/478 (47.3%)	231/478 (48.3%)	
Male factor	111/1068 (10.4%)	94/783 (12.0%)		56/478 (11.7%)	56/478 (11.7%)	
DOR	358/1068 (33.5%)	229/783 (29.3%)		145/478 (30.4%)	127/478 (26.6%)	
Combined	94/1068 (8.8%)	66/783 (8.4%)		35/478 (7.3%)	42/478 (8.8%)	
Unexplained /other	30/1068 (2.8%)	36/783 (4.6%)		16/478 (3.3%)	22/478 (4.6%)	
Insemination method, n (%)			0.061			0.877
IVF	862/1068 (80.7%)	604/783 (77.1%)		372/478 (77.8%)	370/478 (77.4%)	
ICSI	206/1068 (19.3%)	179/783 (22.9%)		106/478 (22.2%)	108/478 (22.6%)	

Date: mean ± SD or (%) (no./total no.). PPOS, progestin-primed ovarian stimulation; LE, letrozole; MPA, medroxyprogesterone acetate; BMI, body mass index; AFC, antral follicle count; AMH, anti-Mullerian hormone; DOR, diminished ovarian reserve; IVF, in vitro fertilization; ICSI, intracytoplasmic sperm injection.

### Ovarian stimulation characteristics

The ovarian stimulation characteristics of the two groups are given in **Table 2**. After PSM, there were significant differences in the dose of MPA, duration of Gn, and number of follicles with diameter > 16 mm on trigger day (*P <*0.05). However, there were no statistically significant differences between the two groups in terms of total dosage of Gn, premature LH surge, endometrial thickness on the hCG trigger day, number of oocytes retrieved, number of mature oocytes, fertilization rate, nonviable embryo cycles, blastocyst progression rate, number of frozen embryos and moderate/severe OHSS rate (*P* > 0.05).

**Table 2:** Ovarian stimulation characteristics of the two PPOS protocols.

	Before propensity matching			After propensity matching		
	LE+ fixed MPA	LE +degressive MPA	*P*-value	LE+ fixed MPA	LE +degressive MPA	*P*-value
Total dosage of MPA (mg)	(1068) 53.6 ± 13.4	(783) 42.6 ± 12.4	<0.001	(478) 52.1 ± 13.1	(478) 44.9 ± 12.5	<0.001
Duration of Gn (days)	(1068) 9.5 ± 1.5	(783) 10.3 ± 1.6	<0.001	(478) 9.7 ± 1.7	(478) 10.3 ± 1.5	<0.001
Total dosage of Gn (IU)	(1068) 1862.2 ± 475.9	(783) 1939.0 ± 604.1	0.177	(478) 1899.9 ± 450.4	(478) 1993.3 ± 597.3	0.260
Premature LH surge (LH > 10mIU/ml)	147/1068 (13.8%)	38/783 (4.9%)	<0.001	30/478 (6.3%)	26/478 (5.4%)	0.582
Endometrial thickness on the hCG trigger day (mm)	(1061) 8.8 ± 2.4	(770) 8.8 ± 2.3	0.441	(475) 9.2 ± 2.5	(470) 8.8 ± 2.3	0.075
No. of follicles with diameter > 16 mm on trigger day	(1063) 5.2 ± 2.9	(776) 6.3 ± 3.1	<0.001	(477) 5.6 ± 3.0	(474) 6.3 ± 3.0	<0.001
No. of oocytes retrieved	(1068) 6.3 ± 2.9	(783) 6.6 ± 2.7	0.031	(478) 6.4 ± 2.9	(478) 6.7 ± 2.7	0.194
No. of mature oocytes	(1068) 5.5 ± 2.8	(783) 5.7 ± 2.6	0.041	(478) 5.5 ± 2.9	(478) 5.8 ± 2.6	0.175
Fertilization rate (2PN) (%)	(1068) 84.0 ± 17.4	(783) 84.3 ± 16.5	0.707	(478) 82.8 ± 17.8	(478) 83.7 ± 16.8	0.428
Cleavage rate (%)	(1068) 98.4 ± 9.3	(783) 98.5 ± 9.4	0.738	(478) 98.2 ± 10.1	(478) 98.3 ± 10.7	0.899
Nonviable embryo cycles	34/1068 (3.2%)	20/783 (2.6%)	0.427	13/478 (2.7%)	8/478 (1.7%)	0.270
No. of viable embryos obtained	(1068) 2.7 ± 1.4	(783) 2.7 ± 1.3	0.332	(478) 2.6 ± 1.4	(478) 2.7 ± 1.3	0.148
No. of top-quality embryos	(1068) 2.0 ± 1.5	(783) 2.0 ± 1.4	0.509	(478) 1.9 ± 1.4	(478) 2.0 ± 1.4	0.126
Blastocyst progression rate (%)	1590/2061 (77.1%)	1482/1814 (81.7%)	<0.001	773/968 (79.9%)	885/1102 (80.3%)	0.797
No. of frozen embryos	(1068) 2.2 ± 1.3	(783) 2.3 ± 1.3	0.133	(478) 2.2 ± 1.3	(478) 2.3 ± 1.3	0.298
Moderate/severe OHSS, n (%)	0	0	/	0	0	/

Date: mean ± SD or (%) (no./total no.). PPOS, progestin-primed ovarian stimulation; LE, letrozole; MPA, medroxyprogesterone acetate; Gn, gonadotropin; LH, luteinizing hormone; PN, pronuclear number; OHSS, ovarian hyperstimulation syndrome.

### Hormone profile

**Figure 3:**
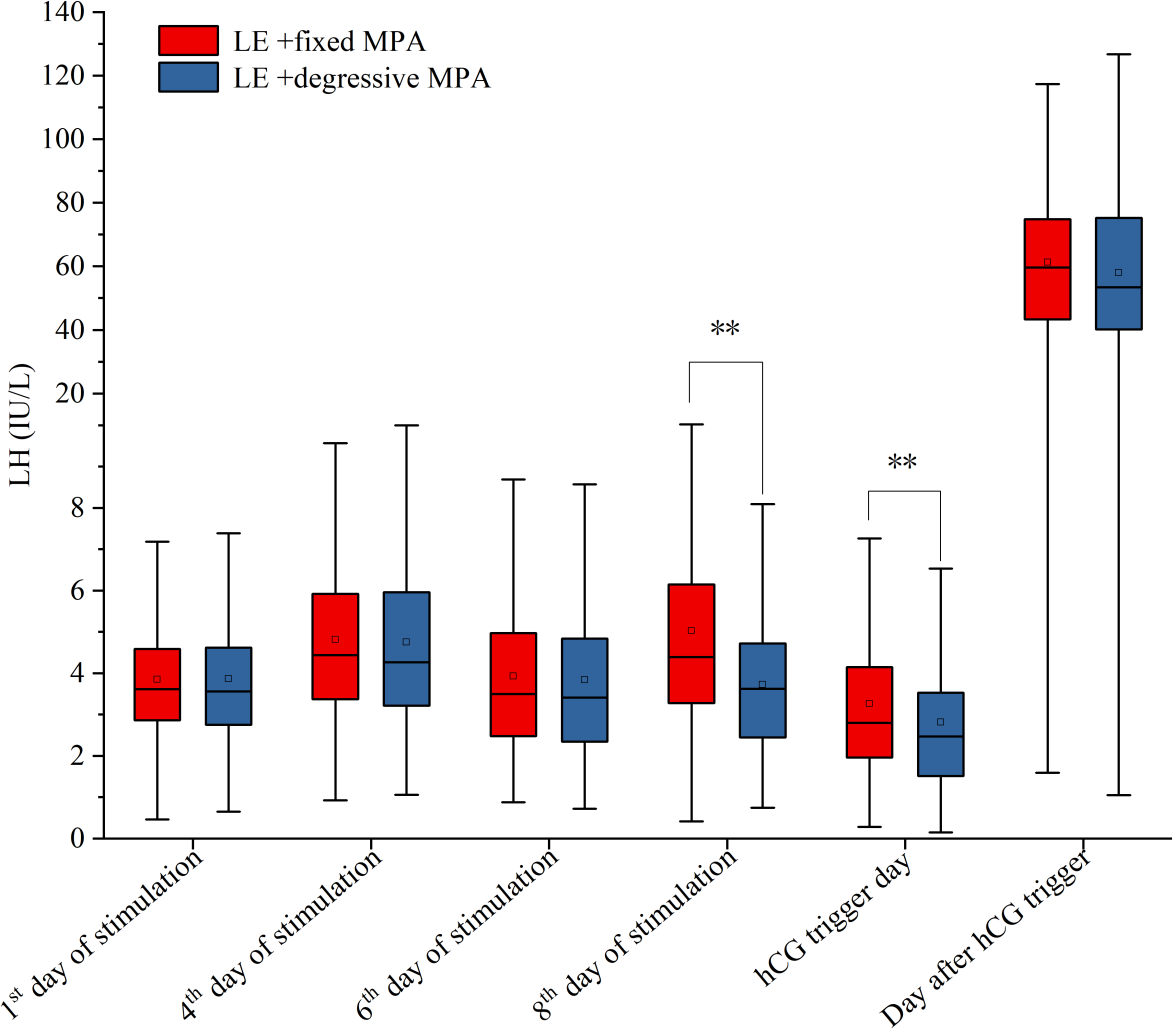
The serum levels of LH on the 1^st^, 4^th^, 6^th^, 8^th^ of stimulation, hCG trigger day and the day after hCG trigger between the two PPOS protocols. **p-value < 0.01.

**Figure 4:**
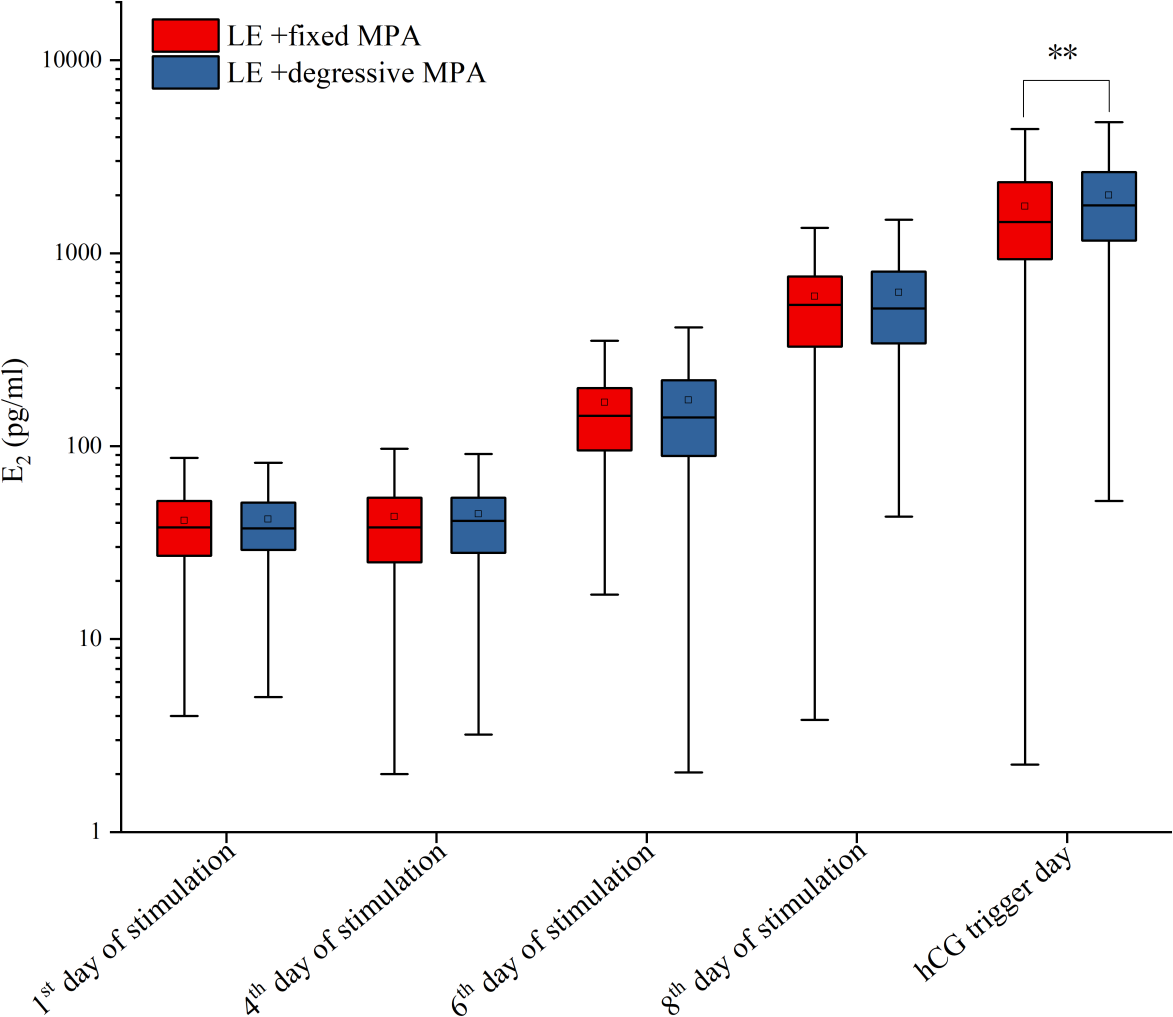
The serum levels of E_2_ on the 1^st^, 4^th^, 6^th^, 8^th^ of stimulation and hCG trigger day between the two PPOS protocols. **p-value < 0.01.

For hormone levels during ovarian stimulation, there were no statistically significant differences in LH and E_2_ levels in the two cohorts on the first day, the fourth day, and the sixth day of stimulation, as well as E_2_ levels on the eighth day of stimulation and LH levels on the day after hCG trigger (*P* > 0.05), but there were significant differences in LH levels on the eighth day of stimulation, and LH, E_2_, and P levels on the hCG trigger day (*P* < 0.01) (**Table 3**; **Figures 3**, **4**).

**Table 3:** Hormone profiles during ovarian stimulation of the two PPOS protocols.

	Before propensity matching			After propensity matching		
	LE+ fixed MPA	LE +degressive MPA	*P*-value	LE+ fixed MPA	LE +degressive MPA	*P*-value
1^st^ day of stimulation						
FSH (IU/L)	(1067) 7.8 ± 1.8	(783) 7.7 ± 1.8	0.158	(478) 7.7 ± 1.8	(478) 7.7 ± 1.8	0.878
LH (IU/L)	(1068) 4.2 ± 1.9	(782) 3.7 ± 1.7	<0.001	(478) 3.9 ± 1.5	(478) 3.9 ± 1.7	0.874
E_2_ (pg/ml)	(1068) 41.5 ± 20.8	(783) 41.4 ± 22.1	0.915	(478) 41.2 ± 21.3	(478) 41.7 ± 20.9	0.697
P (ng/ml)	(1062) 0.6 ± 0.5	(777) 0.6 ± 0.5	0.564	(478) 0.7 ± 0.5	(478) 0.7 ± 0.6	0.937
4^th^ day of stimulation						
LH (IU/L)	(1047) 5.1 ± 2.3	(771) 4.5 ± 2.1	<0.001	(478) 4.8 ± 2.3	(478) 4.8 ± 2.2	0.694
E_2_ (pg/ml)	(1064) 47.1 ± 29.2	(781) 42.4 ± 27.6	<0.001	(478) 42.8 ± 27.0	(478) 44.5 ± 28.7	0.354
6^th^ day of stimulation						
LH (IU/L)	(1015) 5.2 ± 3.1	(680) 3.5 ± 2.0	<0.001	(478) 3.9 ± 2.3	(478) 3.8 ± 2.1	0.548
E_2_ (pg/ml)	(1017) 188.3 ± 140.3	(683) 159.7 ± 124.2	<0.001	(478) 168.6 ± 111.1	(478) 173.4 ± 136.9	0.547
8^th^ day of stimulation						
LH (IU/L)	(813) 5.2 ± 2.6	(378) 3.8 ± 1.8	<0.001	(333) 5.0 ± 2.7	(208) 3.7 ± 1.7	<0.001
E_2_ (pg/ml)	(814) 592.9 ± 441.9	(378) 488.3 ± 422.5	<0.001	(333) 597.9 ± 397.0	(208) 625.9 ± 438.2	0.444
hCG trigger day						
LH (IU/L)	(1065) 3.6 ± 2.5	(778) 2.8 ± 1.9	<0.001	(477) 3.3 ± 2.4	(475) 2.8 ± 1.9	0.002
E_2_ (pg/ml)	(1067) 1708.0 ± 1231.1	(779) 1921.2 ± 1110.4	<0.001	(478) 1752.5 ± 1120.8	(476) 1997.2 ± 1108.5	<0.001
P (ng/ml)	(1019) 0.9 ± 0.5	(777) 0.8 ± 0.4	<0.001	(461) 0.9 ± 0.5	(476) 0.8 ± 0.5	<0.001
Day after hCG trigger						
LH (IU/L)	(864) 65.9 ± 29.3	(695) 56.6 ± 27.0	<0.001	(285) 61.2 ± 29.3	(364) 58.0 ± 27.3	0.150

Date: mean ± SD or (%) (no./total no.). PPOS, progestin-primed ovarian stimulation; LE, letrozole; MPA, medroxyprogesterone acetate; FSH, follicle-stimulating hormone; LH, luteinizing hormone; E_2_, estradiol; P, progesterone; hCG, human chorionic gonadotropin.

### Pregnancy outcomes in frozen-thawed embryo transfer cycles

Descriptive statistics for the reproductive outcomes of frozen-thawed embryo transfer (FET) are summarized in **Table 4**. There was no statistically significant difference between the two groups in the number of transferred embryos, endometrial preparation methods, embryo transfer stage, clinical pregnancy rate (CPR), ectopic pregnancy rate, early pregnancy loss rate, mid- and late-term pregnancy loss rate, live birth rate, CLBR, fetal birth weights, fetal sex, or malformation rate (*P* > 0.05) (**Table 4**).

**Table 4:** Freeze-thaw transplantation cycle and reproductive outcome between the two PPOS protocols.

	Before propensity matching			-	After propensity matching		
	LE+ fixed MPA	LE +degressive MPA	*P*-value		LE+ fixed MPA	LE +degressive MPA	*P*-value
No. of FET	1027	772			459	459	
No. of transferred embryos (per transfer)	(1027) 1.8 ± 0.5	(772) 1.7 ± 0.5	0.003		(459) 1.7 ± 0.5	(459) 1.7 ± 0.5	0.281
Endometrial preparation, n (%)			0.512				0.446
Natural cycle	4/1027 (0.4%)	3/772 (0.4%)			1/459 (0.2%)	2/459 (0.4%)	
HRT	101/1027 (9.8%)	89/772 (11.5%)			39/459 (8.5%)	49/459 (10.7%)	
Down-regulation + HRT	922/1027 (89.8%)	680/772 (88.1%)			419/459 (91.3%)	408/459 (88.9%)	
Embryos transferred n (%)			<0.001				0.084
Cleavage stage	318/1027 (31.0%)	160/772 (20.7%)			116/459 (25.3%)	94/459 (20.5%)	
Blastocyst stage	709/1027 (69.0%)	612/772 (79.3%)			343/459 (74.7%)	365/459 (79.5%)	
Biochemical pregnancy rate, n (%)	677/1027 (65.9%)	521/772 (67.5%)	0.486		310/459 (67.5%)	313/459 (68.2%)	0.832
Clinical pregnancy rate, n (%)	612/1027 (59.5%)	435/772 (56.3%)	0.175		286/459 (62.3%)	270/459 (58.8%)	0.160
Implantation rate, n (%)	807/1808 (44.6%)	559/1305 (42.8%)	0.318		385/797 (48.3%)	356/781 (45.6%)	0.278
Ectopic pregnancy rate, n (%)	8/612 (1.3%)	2/435 (0.5%)	0.286		6/286 (2.1%)	2/270 (0.7%)	0.181
Early pregnancy loss rate, n (%)	89/612 (14.5%)	51/435 (11.7%)	0.283		39/286 (13.6%)	28/270 (10.4%)	0.237
Mid- and late-term pregnancy loss rate, n (%)	17/612 (2.8%)	8/435 (1.8%)	0.327		8/286 (2.8%)	2/270 (0.7%)	0.068
Preterm birth rate, n (%)	118/612 (19.3%)	79/435 (18.2%)	0.648		52/286 (18.8%)	49/270 (18.1%)	0.883
Twin pregnancy rate, n (%)	141/612 (23.0%)	93/435 (21.4%)	0.525		72/286 (25.2%)	62/270 (23.0%)	0.542
Live birth rate, n (%)	490/1027 (47.7%)	373/772 (48.3%)	0.800		229/459 (49.9%)	237/459 (51.6%)	0.403
Cumulative live birth rate, n (%)	490/871 (56.3%)	373/666 (56.0%)	0.922		229/401 (57.1%)	237/413 (57.4%)	0.936
Birth weights (kg)	(627) 2.94 ± 0.67	(466) 2.96 ± 0.66	0.713		(300) 2.92 ± 0.65	(299) 2.94 ± 0.65	0.704
Fetus's sex, n (%)			0.695				0.653
A girl	154/490 (31.4%)	126/373 (33.8%)			73/229 (31.9%)	76/220 (32.1%)	
A boy	198/490 (40.4%)	154/373 (41.3%)			85/229 (37.1%)	99/220 (41.7%)	
Two girls	19/490 (3.9%)	16/373 (4.3%)			11/229 (4.8%)	9/220 (3.8%)	
Two boys	51/490 (10.4%)	37/373 (9.9%)			21/229 (9.2%)	23/220 (9.7%)	
A boy and a girl	68/490 (13.9%)	40/373 (10.7%)			39/229 (17.0%)	30/220 (12.7%)	
Fetal malformation rate (%)	6/490 (1.2%)	6/373 (1.6%)	0.633		4/229 (1.7%)	2/220 (0.9%)	0.440

Date: mean ± SD or (%) (no./total no.). PPOS, progestin-primed ovarian stimulation; LE, letrozole; MPA, medroxyprogesterone acetate; FET, frozen-thawed embryo transfer; HRT, hormone replacement therapy.

To account for potential confounders, multivariable regression analysis was performed. After controlling for female age, BMI, AFC, AMH, duration of infertility, infertility type, infertility diagnosis, fertilization method, serum FSH, LH, E_2_ and P levels on the 1^st^ day of stimulation and serum LH and E_2_ levels on the 4^th^ day and 6^th^ day of stimulation, there were significant differences in total dosage of MPA and number of follicles with diameter more than 16 mm on trigger day (*P* < 0.001) (**Table 5**). Furthermore, there were no significant differences in the premature LH surge rate, number of oocytes retrieved, CLBR or fetal malformation rate after multivariable regression analysis (*P* > 0.05) (**Table 5**).

**Table 5:** Comparison of the correlation between the two PPOS protocols and pregnancy outcomes using multivariable regression analysis before and after propensity score matching.

Exposure	Before propensity matching		After propensity matching	
	Non-adjusted	Adjust I	Non-adjusted	Adjust II
Total dosage of MPA (mg)				
LE + fixed MPA	0	0	0	0
LE + degressive MPA	-11.1 (-12.3, -9.9) <0.001	-7.9 (-9.3, -6.6) <0.001	-7.4 (-9.0, -5.9) <0.001	-6.9 (-8.5, -5.4) <0.001
Premature LH surge				
LE + fixed MPA	1.0	1.0	1.0	1.0
LE + degressive MPA	0.3 (0.2, 0.5) <0.001	0.7 (0.4, 1.3) 0.264	0.8 (0.5, 1.4) 0.473	0.7 (0.3, 1.5) 0.319
number of oocytes retrieved				
LE + fixed MPA	0	0	0	0
LE + degressive MPA	0.3 (0.0, 0.5) 0.031	0.2 (-0.0, 0.5) 0.058	0.1 (-0.2, 0.5) 0.405	0.1 (-0.1, 0.4) 0.276
No. of follicles with diameter > 16 mm on trigger day				
LE + fixed MPA	0	0	0	0
LE + degressive MPA	1.1 (0.8, 1.4) <0.001	0.7 (0.4, 1.0) <0.001	0.7 (0.3, 1.1) <0.001	0.7 (0.4, 1.0) <0.001
Cumulative live birth rate				
LE + fixed MPA	1.0	1.0	1.0	1.0
LE + degressive MPA	1.1 (0.9, 1.3) 0.454	1.0 (0.8, 1.2) 0.837	1.0 (0.8, 1.3) 0.738	1.0 (0.8, 1.3) 0.813
Fetal malformation rate				
LE + fixed MPA	1.0	1.0	1.0	1.0
LE + degressive MPA	1.4 (0.4, 4.3) 0.590	0.9 (0.2, 4.5) 0.900	0.4 (0.1, 2.1) 0.279	0.2 (0.0, 5.6) 0.362

Data was shown as β (95%CI) P value /OR (95%CI) P value.

Non-adjusted model adjusts for: None. Adjust I model and Adjust II model were adjusted for: female age, BMI, AFC, AMH, duration of infertility, infertility type, infertility diagnosis, insemination method, serum FSH, LH, E_2_ and P levels on 1^st^ day of stimulation and serum LH and E_2_ levels on 4^th^ and 6^th^ day of stimulation.

## Discussion

Our study found that the LE + degressive MPA group exhibited lower dosages of MPA, and lower hormone levels (LH and E_2_) during the late follicular stage compared to the fixed 10 mg daily MPA group. Additionally, the LE + degressive MPA group showed a higher duration of Gn and greater numbers of follicles with diameter more than 16 mm on trigger day. However, there were no significant differences between the two groups in terms of premature LH surge, number of oocytes retrieved, moderate/severe OHSS rate, CPR, CLBR, or fetal malformation rate. The use of a degressive MPA dose combined with LE proved effective in reducing the total MPA dosage and promoting follicle maturation in women undergoing the PPOS protocol.

In the current study, three steps were taken to reduce the total dose of MPA. First, we used LE instead of MPA from day 1 to day 3 of ovarian stimulation. Then, on day 4 of stimulation, no MPA or LE was administered. The third step involved administering MPA from day 5 of stimulation until the hCG trigger day, with gradual reduction until complete withdrawal.

LE, a third-generation aromatase inhibitor, promotes folliculogenesis by accumulating androgen in the follicle while increasing FSH receptor expression and stimulating insulin-like growth factor-I (IGF-I) (21, 22). Notably, LE treatment in women with PCOS resulted in a trend of monofollicular growth in the late follicular stage (23). Two retrospective studies on PCOS patients using a combination of LE and MPA in IVF cycles reported a higher follicular output rate (24) without compromising mature and fertilized oocyte yields, despite decreased oocyte maturity and fertilization rates (13). These studies used LE for at least five days, similar to the 5-day clomiphene citrate (CC) regimen for ovulation induction (21). However, some research has shown that a single dose of LE (20-25 mg) on day 3 of the cycle or a 5-day LE regimen yields similar reproductive outcomes (25, 26), suggesting possibilities for reducing the LE usage days. To ensure multiple follicular development while preventing monofollicular growth, this study employed a 3-day LE treatment. Adding LE to Gn has been shown to effectively lower Gn requirements in previous reports (21). In our study, we adopted a sequential application of LE and MPA instead of simultaneous use, which our team previously found effective in patients with normal ovarian reserve (14). This approach allows for a reduction in the MPA dose and initial Gn dose, leading to cost savings during ovarian stimulation. Additionally, LE has a mean half-life of approximately 45 hours and is quickly reversible after discontinuation (21). Thus, abstaining from the administration of LE and MPA for approximately 2 days after the 3-day LE treatment provides another feasible strategy for decreasing the MPA dose.

There are two crucial aspects of MPA administration: dosage and timing. The inhibitory effect on an untimely LH increase can be determined by considering both factors. While a previous study by Wikström et al. (27) demonstrated that a 5 mg MPA dose failed to suppress ovulation, recent research with varied MPA doses, such as 4 mg, 6 mg, and 10 mg daily, proved effective in preventing premature LH surges (3, 6, 12). Hence, our presumption is that the MPA dosage used in IVF cycles is less critical than the precise timing of its administration. To achieve optimal results, MPA should be applied before the LH surge induced by E_2_ (10). As a flexible-start MPA protocol, the initiation of MPA usage could occur on stimulation day 7 or when the leading follicle reaches ≥ 12-14 mm or serum E_2_ levels reach > 200 ng/mL (5, 28–33). Notably, the peak plasma MPA concentration is typically reached 1-3 hours after oral administration (34), and the pituitary LH levels decrease after 5 days of MPA administration (10). Furthermore, it takes three weeks or longer for serum LH levels to recover after oral intake of 10 mg MPA per day for 10 days (35). In our study, we administered MPA on stimulation day 5, which is earlier than the timing mentioned in the literature. We also adopt a degressive administration approach for MPA, based on stable serum LH levels, preventing delayed resumption of LH levels. Our findings suggest a promising beneficial effect, as it allows for a reduction in MPA dosage while ensuring effective pituitary suppression.

Emphasis should be placed on the impact of LH on various stages of follicle growth. A study confirmed that elevated basal LH levels in PCOS patients undergoing IVF treatment with the MPA protocol do not impair pregnancy outcomes (36). To ensure optimal follicle development in IVF cycles with suppressed endogenous LH, LH supplementation is recommended when basal LH levels are less than 1.2 IU/L (37). Furthermore, different stages of follicle development are influenced by distinct survival factors for follicle growth. Although during the antral follicle stage, FSH plays a major role as a survival factor, while IGF1 and IL1b act as potent survival factors (38), elevated LH levels after LE treatment could potentially serve as a predictor for improved ovulation induction outcomes and no need for preinhibition of LH secretion (39). In preovulatory follicles of middle and late follicular stages, both FSH and LH play crucial roles as survival factors (38). Therefore, if the serum LH levels of the ovarian stimulation process remain stable, adopting MPA later than early follicular stage and administration degressively is considered safe.

Previous studies have reported varying LH levels on the hCG trigger day in different patient groups using the MPA protocol. In women with PCOS, LH levels ranged from 1.62 to 2.52 IU/L (40–43), while in infertile women with normal ovarian reserve, LH levels were between 1.56 and 3.54 IU/L (12, 14, 44–46). Poor responders showed LH levels in the range of 2.4 to 5.55 IU/L (6, 47–49). Moreover, research indicated that the LH level at the hCG trigger was 3.68 ± 2.69 IU/L for patients younger than 35 years and 4.77 ± 3.10 IU/L for patients older than 35 years (49). Although the suitable values for LH levels on the hCG trigger day require further investigation, it appears that they are positively correlated with age and negatively correlated with ovarian reserve. In this study, the LH level on the hCG trigger day was lower in the LE + degressive MPA group than in the LE + fixed MPA group, suggesting that individualized degression could effectively result in ovarian suppression without affecting ovulation and pregnancy outcomes.

When assessing the efficacy of MPA in pituitary suppression, the incidence of a premature LH surge serves as a crucial indicator for evaluation. In PCOS patients, no cases of premature LH surge were reported (41, 43), while normal responders among infertile women had an incidence of 0-0.7% (10, 12). Studies on poor responders revealed a range of 0.6%-5.6% premature LH increase (3, 48, 50). These findings suggest a negative correlation between the incidence of premature LH surge and ovarian reserve; however, further investigations are required to establish strong and direct evidence. In our study, we observed a comparable occurrence of premature LH increase during the middle to late stage of follicular growth in the LE + fixed MPA group compared to the LE + degressive MPA group (6.3% vs 5.4%), with no cases canceled in either group. Therefore, we presume that the MPA degressive regimen has an efficiency on pituitary suppression.

It is crucial to consider the potential impact of MPA on oocyte quality, and consequently, embryo quality and fetal growth, during the administration process. Despite some case series reporting adverse reproductive development after *in utero* exposure, there are reassuring findings regarding neonatal outcomes following MPA usage in a collection of retrospective studies (51–53). In accordance with these results, our study revealed no significant difference in reproductive outcomes and fetal malformation rates, leading to the conclusion that MPA at a daily dose of 10 mg for approximately 10 days or less appears to be relatively safe.

To our knowledge, this is the first study aimed at evaluating the efficacy of a step-by-step reduction in MPA dosage compared to a daily 10 mg dose in IVF/ICSI patients with PPOS protocols, focusing on endocrinological characteristics and clinical outcomes. This novel approach offers valuable insights to improve the regimen for PPOS ovarian stimulation. Another notable strength of our study is the implementation of PSM analysis, which helps mitigate bias in this retrospective cohort study. Additionally, this study benefits from a relatively large sample size, encompassing a diverse population aged between 20 and 40 years, providing meaningful representation of women facing infertility. Furthermore, recording neonatal outcomes adds to the credibility and reliability of this study.

However, this study has several limitations that should be acknowledged. First, its retrospective nature calls for further validation through randomized controlled trials and multicenter studies to confirm the results. Second, the study population from our reproductive center had a higher average age and lower AFC than other research, potentially limiting the generalizability of the findings to younger women, PCOS patients, or other specific groups of infertility patients. Additionally, the administration of different stimulation drugs (recombinant FSH and HMG) and flexible initial Gn doses may have influenced the hormonal outcomes, adding a degree of complexity to the analysis. While the CLBR was utilized as a recommended measure for evaluating IVF/ICSI treatment outcomes, it is worth noting that 308 and 355 embryos were still awaiting transfer in the LE + fixed MPA and LE + degressive MPA groups, possibly affecting the precision of the conclusion.

## Conclusion

This retrospective study demonstrates the effectiveness of degressive MPA combined with LE in reducing the total MPA dose without compromising the stimulation outcomes in IVF patients. This approach offers advantages such as cost-effective stimulation, personalized treatment, and comparable reproductive outcomes. To validate the practicality of this regimen and to determine the optimal LH level and initial Gn dose for IVF, further prospective randomized controlled trials are warranted.

## Data availability statement

The original contributions presented in the study are included in the article/**Supplementary Material**, further inquiries can be directed to the corresponding author/s.

## Ethics statement

The studies involving humans were approved by This study was performed in accordance with the principles of the Declaration of Helsinki and was approved by the Ethics Committee of Renmin Hospital, Hubei University of Medicine (No: syrmyy2023-051). The studies were conducted in accordance with the local legislation and institutional requirements. The participants provided their written informed consent to participate in this study.

## Author contributions

CZ: Conceptualization, Project administration, Resources, Supervision, Writing – review & editing. YZ: Funding acquisition, Methodology, Project administration, Resources, Supervision, Writing – original draft, Writing – review & editing. HL: Data curation, Formal Analysis, Investigation, Software, Visualization, Writing – original draft, Writing – review & editing. SZ: Data curation, Writing – review & editing. SJ: Data curation, Writing – review & editing. WZ: Data curation, Writing – review & editing. XW: Data curation, Writing – review & editing. LT: Data curation, Writing – review & editing. GZ: Data curation, Writing – review & editing. NH: Data curation, Writing – review & editing. HD: Project administration, Supervision, Writing – review & editing. HC: Project administration, Supervision, Writing – review & editing.
